# ‘On the same level’: facilitators’ experiences running a drug user-led safer injecting education campaign

**DOI:** 10.1186/1477-7517-10-4

**Published:** 2013-03-06

**Authors:** Cody Callon, Grant Charles, Rick Alexander, Will Small, Thomas Kerr

**Affiliations:** 1 British Columbia Centre for Excellence in HIV/AIDS, St. Paul’s Hospital, 608-1081 Burrard Street, Vancouver, BC V6Z 1Y6, Canada; 2 School of Social Work, University of British Columbia, 2080 West Mall, Vancouver, BC, V6T 1Z2, Canada; 3 Department of Medicine, Faculty of Medicine, University of British Columbia, 2194 Health Sciences Mall, Vancouver, BC V6T 1Z3, Canada; 4 Vancouver Area Network of Drug Users, 380 East Hastings Street, Vancouver, BC, V6A 1P4, Canada

**Keywords:** Injection drug use, Safer injecting education, User-led intervention

## Abstract

**Background:**

Unsafe injection practices play a major role in elevated rates of morbidity and mortality among people who inject drugs (IDU). There is growing interest in the direct involvement of IDU in interventions that seek to address unsafe injecting. This study describes a drug user-led safer injecting education campaign, and explores facilitators’ experiences delivering educational workshops.

**Methods:**

We conducted semi-structured qualitative interviews with 8 members of the Injection Support (IS) Team who developed and facilitated a series of safer injecting education workshops. Interviews explored facilitator’s perceptions of the workshops, experiences being a facilitator, and perspectives on the educational campaign. Interviews were transcribed verbatim and a thematic analysis was conducted.

**Results:**

IS Team facilitators described how the workshop’s structure and content enabled effective communication of information about safer injecting practices, while targeting the unsafe practices of workshop participants. Facilitators’ identity as IDU enhanced their ability to relate to workshop participants and communicate educational messages in language accessible to workshop participants. Facilitators reported gaining knowledge and skills from their involvement in the campaign, as well as positive feelings about themselves from the realization that they were helping people to protect their health. Overall, facilitators felt that this campaign provided IDU with valuable information, although facilitators also critiqued the campaign and suggested improvements for future efforts.

**Conclusions:**

This study demonstrates the feasibility of involving IDU in educational initiatives targeting unsafe injecting. Findings illustrate how IDU involvement in prevention activities improves relevance and cultural appropriateness of interventions while providing individual, social, and professional benefits to those IDU delivering education.

## Background

Injection drug use is a growing public health concern, due to the morbidity and mortality observed among people who inject drugs (IDU) [1-5]. IDU are vulnerable to an array of health related harms, including but not limited to HIV, hepatitis C, bacterial and fungal infections, and venous damage [3,6-9]. Many of these health harms are the result of unsafe injection practices, which are preventable given proper preparation and administration of drugs by injection.

In response to increased concern for the health and well being of IDU, a number of intervention and prevention strategies have been implemented to address the harms stemming from injection drug use. Most prominent among these are distribution of sterile syringes and injection paraphernalia [10-12], harm reduction outreach programs [13,14], medically supervised injection facilities [15-17], and educational and behavioural interventions [18-20]. Educational materials such as posters and pamphlets are a mainstay of educational programs, and many public health initiatives are founded on the assumption that IDU lack knowledge regarding correct injection procedures [21,22]. However, there is a pronounced lack of evidence-based evaluation of these materials. Despite implementation of all of the aforementioned intervention and prevention strategies in Vancouver Canada, research shows that high rates of unsafe injecting persist [23-26].

Given ongoing injection related problems, there is a growing interest in the involvement of IDU in initiatives that address unsafe injecting practices and the associated adverse health consequences and costs. Researchers in a number of settings internationally have implemented peer-driven network oriented HIV prevention interventions in which public health experts train peer leaders to disseminate prevention information and supplies through micro- and macro- networks of IDU [27-32]. Evaluation of these approaches highlights several advantages of peers over traditional outreach methods. Peers are often viewed as more credible and influential sources of information [27,28,33], and have the ability to use already established networks to reach more hidden and diverse populations of IDU [27,28,33,34]. Furthermore, peers are able to reach IDU with prevention information and supplies in places and at times when high-risk behaviours are most likely to occur [27,28,33], and peer-based education is often more cost effective than traditional outreach programs [27,28].

Much less is known about the effectiveness of interventions that have been developed by IDU, which have emerged from drug-user led efforts to address the harms associated with unsafe injecting. Although descriptions of user-led organizations of IDU in Europe and Australia are available in the literature [35-38], there are few evaluations of the educational initiatives developed by these groups. Evaluations of the Mitsampan Harm Reduction Centre operated by the Thai Drug Users Network in Bangkok, Thailand, and programs organized by the Vancouver Area Network of Drug Users in Vancouver, Canada, show that these user-led initiatives extend the reach and effectiveness of harm reduction services available in these areas [39-43].

Given the remarkable lack of description and evaluation of user-led approaches to reducing the harms associated with injection drug use, and complete absence in the literature of the perspectives of IDU that lead user-led interventions, we conducted a qualitative exploration of facilitators’ experiences leading a user-led safer injecting education campaign.

### The VANDU Injection Support Team

The Vancouver Area Network of Drug Users (VANDU) is active in direct action and advocacy, and has run a variety of drug user-led programs, including needle distribution and recovery, an outreach-based alley patrol program, as well as various action, education, and support groups [40]. In response to the harms associated with assisted and unsafe injection practices, VANDU expanded their alley patrol program in August 2005 to develop an ‘Injection Support Team’ (IS Team), which facilitates education and engagement with large numbers of IDU. The IS Team is composed of nine current and former IDU who are recognized as ‘hit doctors’, individuals who are regularly asked to provide assistance with injections, within the local injecting scene. This group of individuals initiated the IS Team on a volunteer basis in response to the ongoing harms associated with unsafe and assisted injecting, as well as the prohibition on assisted injections at the supervised injection facility. Following the establishment of the IS Team, a community-based research project was initiated in partnership between VANDU, the IS Team, and the British Columbia Centre for Excellence in HIV/AIDS. The IS Team developed the following mission statement emphasizing their basic purpose and activities:

The VANDU IS Team is a user-led program that provides peer-to-peer education and assistance to promote safer injecting practices. Through advocacy and outreach the IS Team seeks to reduce the harms resulting from unsafe injection and preserve the health of injection drug users.

Although the IS Team began as an outreach-based program, there was ongoing interest among the IS Team members in developing a safer injecting education curriculum that could be delivered in a group setting. When the IS Team decided to undertake this curriculum development process, all IS Team members were invited to participate, and only one member declined the opportunity. Curriculum development and workshop facilitation responsibilities were then divided amongst the IS Team members based on their individual interest and expertise.

### The IS Team education campaign

Utilizing a participatory education approach [44,45] drawing on research findings, outreach activities, and community consultation, the IS Team developed a curriculum, training materials, and novel demonstration processes for five 90 minute workshops promoting safer injecting practices. The IS Team education campaign was implemented between November 2009 and April 2010 at five separate locations in Vancouver’s Downtown Eastside (DTES) including, the VANDU storefront office, two NGO operated single room occupancy hotels, and two public drop-in centres.

The first workshop in this campaign described various unsafe injection practices (e.g., sharing syringes and other paraphernalia, jugular injections, and assisted injection), provided an overview of the history, training, and outreach activities of the IS Team, and outlined the format and content of upcoming workshops. The second workshop focused on the consequences of unsafe injecting including, information on how bacteria gets introduced during the injection process, adulterants and additives commonly contained in illicit drugs, and description of the viral infections, venous damage, and bacterial infections resulting from unsafe injection practices. This workshop concluded with a discussion of the locations where participants can access relevant healthcare services. The third workshop involved step-by-step hands on demonstrations and practice of injecting skills and techniques concurrent with a discussion of why each step is important. Common barriers and challenges to safe injecting (e.g., lack of supplies, not having a safe place to inject, and police surveillance and interference) were discussed, as were strategies that can be used to inject safely more often. The fourth workshop provided step-by-step demonstrations on how to correctly prepare various drugs common in the local context including heroin, cocaine, crack, methamphetamine, and a range of diverted pharmaceuticals^1^ for injection. The final workshop covered transmission and prevention of HIV and hepatitis C, attempted to dispel common myths about these diseases, and provided information about where testing, treatment, and other services are available.

Each workshop was facilitated by three IS Team members. The IS Team education campaign utilized a mixture of visual learning materials, including PowerPoint slides, poster sized images, and handouts, to visually communicate step-by-step processes, various symptoms of illnesses, and modes of disease transmission. In workshops involving demonstrations, facilitators divided participants into three smaller groups where they discussed, demonstrated, and supervised proper technique and procedure for preparation and administration of injections. IS Team members’ facilitation style involved a participatory education approach in which facilitators and workshop participants engaged as equals and co-learners in the education process. Drawing on the collective knowledge and experiences of each group, facilitators provided contextually appropriate safer injecting education while using real life stories and scenarios to stimulate broader discussion of relevant myths as well as the challenges and barriers to safer injecting practices. Within workshops, facilitators aimed to promote mutual support based on shared experience and equality, while emphasising a harm reduction approach focused on caring and self-preservation. Facilitators were compensated $20 (CAD) for each workshop and workshop participants received $3 (CAD) for their participation. Compensation rates were openly negotiated between the IS Team members and the VANDU Board of Directors, and were consistent with the compensation structure used at VANDU.

## Methods

This project utilized a community-based research approach [46,47] involving active collaboration with members of the IS Team throughout the planning, development, and implementation of the project. A member of the research team (CC) attended all planning and development meetings related to the IS Team education campaign, observed all IS Team education campaign workshops, and actively sought team member’s opinions and feedback on research process and emerging themes.

In-depth interviews were conducted with IS Team members to discuss their perceptions of the workshops they facilitated, their experiences being a facilitator, and their perspective on the overall IS Team education campaign. All IS Team members who facilitated education campaign workshops were invited to participate in an interview. All interviews lasted between 50 and 80 minutes and were conducted between June and August 2010.

Interviews were facilitated using a semi-structured topic guide to encourage discussion of how IS Team members viewed the format and content of the education campaign, how this compared to their previous experiences with safer injecting education, how they perceived their role within the campaign, and perspectives on facilitating drug user-led safer injecting education. All interviews were audio-recorded and transcribed verbatim. A qualitative descriptive methodology was employed for the analysis of these interviews [48]. Analysis began with a detailed open coding of transcripts and theoretical memoing in Atlas.ti software, then validated through systematic review using a constant comparative analysis [49,50]. One member of the research team (CC) was responsible for conducting all interviews and coding all transcripts. Emergent themes and relevant excerpts from the interviews were shared and discussed with all IS Team members to ensure accuracy of interpretations.

Every IS Team member interviewed provided informed consent to participate, and the study was undertaken with appropriate ethical approval granted by the Providence Healthcare/University of British Columbia Research Ethics Board. IS Team members were compensated for their time in the research interview with a $20 (CAD) honorarium. There were no refusals of the offer to participate in the interview, and no drop-outs occurred during the interview process.

## Results

In total 340 unique individuals attended IS Team education workshops, including 157 (46%) women, 177 (52%) men, and 6 (2%) transgendered individuals. IS Team members who participated in qualitative interviews included 3 females and 5 males. The median age of IS Team members was 47.5 (range = 35–59 years). The IS Team included a mix of Caucasian and Aboriginal members as to reflect the demographic of the local drug using population. One of the nine IS Team members was not asked to participate because he withdrew from the team early in the development phase of this education campaign. Representative excerpts from the qualitative interviews are presented below in order to illustrate the central themes that emerged in the analysis. Considerable overlap was observed across thematic areas.

### Format and content of IS Team educational workshops - ‘it taught the nitty gritty of what you’re doing’

IS Team facilitators described a number of aspects of this education campaign that contributed to their ability to communicate information about safer injecting practices while identifying and addressing the unsafe practices of workshop participants. Facilitators expressed that an important aspect of the curriculum was that it went beyond the mechanics of safe injection, to describe why each step is important, and the consequences that can arise from incorrect implementation.

*That’s why our workshops were so successful, because it taught the nitty gritty of what you’re doing and what you’re doing wrong … tell ‘em why it’s not right you know, and make it so the right thing fits into their ritual. Not to stop doing it, but make it fit into their ritual.* (Female Facilitator #1)

The participatory and interactive nature of these workshops was seen as a critical factor in engaging participants. This enabled discussion of the realities of individual’s injecting rituals, the context in which they use, and the strategies that can be implemented to improve injecting practices.

*You could tell some people got really jazzed when they were talking about personal experiences and stuff. Which I thought was a really good thing … because people’s own experiences, that’s what it’s all about right? … Cause we’re talking about it from a, this is the way it should be, not necessarily the way it is, and they’re talking about it from the way it is. So I think it was really important.* (Male Facilitator #2)

An example of a common barrier to enacting risk reduction practices frequently identified within workshops was the inability to access sterile cookers necessary to correctly prepare drugs for injection. IS Team facilitators emphasized the importance of always mixing and filtering drugs prior to injection, and a common solution they proposed was to carry a metal spoon or use the concaved bottom of a beverage can for mixing, while always being sure to disinfect the preparation surface with an alcohol swab.

Most facilitators reported that facilitating with two other IS Team members was a strength of the education format. Working in groups of three created structure among facilitators, while allowing them to educate based on their topics of expertise, and enhancing opportunities to incorporate stories with their explanations.

*I was the more structured one and he was the more off the cuff type of guy. Like when questions came up and stuff he had more information because of his experience.* (Male Facilitator #2)

The use of various forms of education materials and different teaching styles were described as major strengths of the campaign because it engaged a wide range of participants with different learning needs. It is notable that the images utilized were described as particularly useful for communicating the severity of the consequences of unsafe injecting, and assisted facilitators in maintaining their focus when interacting with workshop participants.

*The shock value of the pictures and then being able to note how not to let that happen to yourself, it was really good … it also gave us something to focus on … if we got lost on something we could just turn to the pictures.* (Female Facilitator #1)

### Shared identity of facilitators and participants - ‘in the same sort of head space’

All IS Team facilitators expressed that their knowledge and experience as IDU fostered a sense of shared identity and equality with workshop participants, which encouraged trust and rapport. This also allowed them to present safer injecting information with examples and language that was appropriate and easy to understand.

*The people that were facilitating it and the people that were the members, they’re more or less on the same level. So you could understand,* [you] *don’t have to ask questions or why they didn’t get that, you just know right. Cause you’re both in the same sort of head space, you’re street people right.* (Male Facilitator #3)

Many facilitators described their familiarity with workshop participants as increasing their credibility and making participants more comfortable asking questions or sharing information.

*I sound like I’m just talking with buddies cause I’m just sitting there talking to people that I know … And there’s the other part of it, I’m not at the front of the room because I’m more important than you, I’m just the one that’s doing most of the talking, but you guys kick in when you can cause you guys want answers as do we, and I’m trying to draw them out and get them to participate.* (Male Facilitator #6)

Facilitators identified how having IDU as facilitators minimized power dynamics that frequently exist between educators and learners. Facilitators expressed that their approach differed from their previous experiences receiving safer injecting information.

*We’re them, it’s not like we’re gonna preach to them … Most of them* [formal educators] *kind of seem older or else like they’re trying to talk at them, talk down to them. It’s more of a classroom type way they talk instead of talking like friends or drug users talk to each other, the same way we’re talking … You can pick up more that way cause you don’t feel like you’re being lectured to.* (Male Facilitator #4)

### Facilitator’s personal gains - ‘it was a really good experience for me’

IS Team facilitators articulated various personal gains from their involvement with the campaign, as most expressed that facilitating these workshops improved their overall confidence and public speaking skills. Facilitators also expressed that workshops enhanced interpersonal social skills and network connections, as well as changing their attitude towards dealing with other organizations and professionals.

*I’d gotten into pretty much a mode where I’d just hang out by myself so I didn’t really talk to anybody. This … got me back on meeting people, and talking to people, and realizing the common ground with other people in the community.* (Male Facilitator #7)

*I have developed a more professional attitude when dealing with professionals. I’m more polite, courteous, well-spoken, time to listen. I’ll do everything but wear the tie and nametag.* (Male Facilitator #6)

Facilitators also described the positive feelings they gained from the realization that they were making a difference by helping people to protect their health.

*The idea of imparting knowledge to people that is hopefully going to do something positive for them, I mean, what’s better than that … Especially when you know there’s a need for it. … Like I said, when I first got into it, it was just for the financial thing, but once I got into it, knowing that you’re actually making a difference, that you might actually help someone or something, that’s a good feeling. I’ve never done that type of thing, so it was a really good experience for me.* (Male Facilitator #2)

A few facilitators reported the added benefit of making changes to their own injecting practices based on information they learned through the development and implementation of the campaign. For example, facilitators reported using alcohol swabs and ties more consistently and avoiding the re-use of their own syringes.

### Campaign impact - ‘it works … it’s better’

IS Team facilitators overwhelmingly felt that this campaign provided participants with valuable safer injecting information, some of which including information on prevention of bacterial infections and how to correctly prepare various drugs for injection, is not currently available anywhere else. Most facilitators articulated that educational workshops not only change injection practices, but also have the added benefit of connecting IDU with VANDU and other health services.

*I hear it from people every day. Every day somebody comes up to me and says something. Oh, I tried this, I tried that. It works, it works, it’s better. Can I go to treatment? Where do I go to treatment? You know just questions that were all brought up from those workshops. And it brought a lot of people to VANDU.* (Female Facilitator #1)

Although all IS Team facilitators spoke of the value of providing IDU with accurate information about preparation and administration of injections, they also emphasized numerous contextual barriers and challenges that can make injecting safely difficult. Most commonly, facilitators identified not having a safe place to inject and fear of the police as the predominant contextual factors that can make it difficult to implement safer injecting knowledge.

*If people don’t have a home, if they don’t have a sterile place, if they’re forced to try to hide in a back alley, or in a bush, or some other place where they can’t be found cause they’re so scared of cops. How do you expect any part of that to be clean or safe?* (Female Facilitator #8)

Given the limits of education in addressing contextual factors that perpetuate unsafe injecting, facilitators articulated a desire to expand and improve their activities to pursue broader change beyond education. Their suggestions predominately focused around opening a user-led facility and further expanding their outreach activities.

*Just a separate spot off the VANDU property that people could come to get assisted injection and have a coffee, you know stuff like that … A safe* [inhalation] *site, a safe injection site, that’s not run by government frigging employees.* (Male Facilitator #7)

Numerous facilitators suggested that it would be beneficial to expand their outreach activities beyond provision of harm reduction supplies and safer injecting education to assist with finding housing, provision of lifeskills, assistance finding employment, and support in accessing healthcare and addictions services.

### Criticisms and suggestions - ‘I wanted to do more’

Criticisms of the campaign varied from facilitator to facilitator based on their experiences and the specific workshops they facilitated. A number of the facilitators identified other facilitators arriving at workshops sick, or not showing up, as major issues that impacted the rest of the facilitator’s ability to properly cover workshop curriculum.

*Sometimes she would be sick when she came in, and she wasn’t very together, so that’s when I’d have to prolong the workshop I was doing.* (Female Facilitator #1)

*A few people didn’t show up to do it … they’d go and do something else.* (Male Facilitator #7)

A couple facilitators also indicated that sharing of misinformation was a problem that sometimes occurred during workshops when facilitators deviated from facts to opinions.

*She was giving her own personal opinion on things that weren’t actually proven to be true and we don’t wanna give that kind of information out.* (Female Facilitator #1)

Although facilitators spent a significant amount of time developing workshop curriculum, no time was spent prior to the implementation of the campaign developing the facilitation skills of the IS Team members. A few facilitators noted that managing a large group of IDU can be difficult, and that they would have benefited from more time spent developing facilitation skills before starting the campaign.

*I’d of had better preparation for the facilitators … maybe it would be a practice class. I don’t know how to solve the problem.* (Male Facilitator #6)

Facilitators noted that information on overdoses was missing from the curriculum and could have been added to strengthen the overall campaign.

*I wanted to do more things to let people know to give mouth-to-mouth if your buddy goes down* [overdoses] *in a hotel room. Cause through Christmas time we lost a few people just because they didn’t breathe. Nobody gave them mouth-to-mouth.* (Female Facilitator #5)

Culture and gender specific issues were also identified as important types of information that were missing from the curriculum. While information regarding issues commonly experienced by female injectors (e.g., assisted injection) was included in the workshops, some felt that it would be beneficial to incorporate increased emphasis on particular elements to better address gender-specific educational needs.

*There’s issues that women deal with that are different from men. Like maybe they don’t know how to inject because they’ve always been hit* [injected] *by their boyfriend … With the women you can have information based on jugging* [injecting into the jugular vein] *and why this is more common … more information on the different reasons people use, you know women are more affected by emotional things, we could talk about that.* (Female Facilitator #8)

Similarly, this facilitator suggested that these workshops might have had a greater impact on Aboriginal IDU if specific information was included on the high rates of injecting related morbidity among this sub-population.

*I think you could stress what huge a problem this is for the Aboriginal community … maybe they’re not aware of how serious and how terrible a thing this is … and maybe by educating them they would become more concerned and maybe it would change things. (*Female Facilitator #8)

One of the facilitators of the HIV and hepatitis C workshop felt that they were not fully prepared to respond in-depth to participants’ questions about these diseases and treatments available.

*It should’ve been two workshops. One on hep C and one on HIV because I don’t know all that much about HIV … And maybe a nurse with it because there’s lots of HIV facts and stuff that you need a nurse to tell you about, your medications and stuff, that I just wasn’t prepared for.* (Female Facilitator #1)

Facilitators of this particular workshop felt that it could have been improved by incorporating the technical expertise of a professional educator alongside IS Team members.

## Conclusions

In this study, facilitators described how aspects of IS Team workshop structure and content, including the participatory approach, facilitating in groups, and the variety of educational materials used, helped facilitators communicate information about safer injecting practices while addressing the realities of participant’s injecting rituals. Facilitators felt that their knowledge and experience as IDU increased their credibility and allowed them to communicate with workshop participants in clear and understandable ways. Most IS Team facilitators reported gaining knowledge, skills, and positive feelings about themselves from their involvement in this education campaign. Overall, facilitators felt that this campaign provided IDU with valuable and necessary safer injecting information, however facilitators also provided criticisms and suggestions for future improvement.

The findings that facilitators’ experiences as IDU increased their credibility as educators is consistent with other studies demonstrating that knowledge from personal experience and trust are important aspects of peer interventions, which contribute to greater credibility and influence over behaviour change [27,33,37,40]. Given considerable local variation among cultures and histories of IDU, researchers have argued that successful interventions targeting this population need to involve IDU with extensive knowledge and local experience [40,51]. Previous research on interventions involving IDU as educators and outreach workers suggests that these individuals have the most knowledge and best information about the experiences and current practices of IDU [27,28,37,40]. This is supported by study results indicating that IS Team facilitators utilized their existing knowledge of commonly occurring unsafe injecting practices to identify mistakes made by local IDU during preparation and administration of injections. Based on their own experiences, facilitators were able to adopt a pragmatic approach to discussing various injection practices by identifying why each step is important, potential consequences of unsafe practice, and discussing the contextual factors that can make following these steps difficult, as well as the strategies that can be used to address them. Through their focus on common barriers to safer injecting and navigating common situations experienced by local IDU, the IS Team campaign not only expands the reach of safer injecting education, but also incorporates novel elements which are not currently available elsewhere. Furthermore, examinations of peer-driven interventions from other settings have shown that IDU involvement provides built-in accommodation to the cultural and ethnic diversity of the IDU population by couching prevention and intervention messages in locally appropriate terms [28,52,53]. The results of this study further these findings by showing that IS team facilitators were able to communicate and share information in a language accessible to workshop participants, and were successful in drawing from their own experience to enable discussions of the realities of participants’ injecting rituals. Facilitators emphasized that this mutual understanding minimized unequal power dynamics often existing between educators and learners, which have been identified by other researchers as being counterproductive to educational goals [37,52].

Previous research has found that peer involvement in prevention and advocacy work leads to positive identity and pro-social role development, engendering a sense of purpose and self-respect among IDU, which contrasts the stigma often imposed on them by society [40,53,54]. The present study supports these findings as most facilitators reported developing social and professional skills as a result of their involvement in IS Team workshops. Furthermore, many facilitators described the positive feelings they gained from realizing that they were making a difference in their community by helping people to protect their health. This is consistent with research on motivation among peer workers showing that concern for one’s own community and gaining satisfaction from helping others are major motivators for conducting peer prevention work [27,53]. Facilitators also reported improving their own injection practices following the IS Team education campaign. This is consistent with examinations of peer leaders within larger social network interventions showing that peers involved in education and outreach report the greatest reductions in injection risk behaviours after a follow-up period [27,28,30,53,54]. The results of this study suggest that employing non-users in prevention and intervention work restricts IDU from receiving the aforementioned benefits of this type of work, while evidence indicates that they may in fact be the most suitable candidates to deliver educational messages.

Results of the present study indicate that the IS Team education campaign provided participants with culturally appropriate safer injecting education that addressed issues relevant to local IDU. In this way, the IS Team continues a tradition of education and support programs at VANDU that meet the immediate needs of IDU locally [40,41,43]. This is consistent with research on the harm reduction and prevention activities of user groups and IDU in other settings demonstrating that IDU are capable of active participation in their individual and collective health and often develop novel interventions that extend the range of existing services [38-41,51,55]. Facilitators’ experiences in this campaign also raised a few practical considerations for the development and delivery of these types of programs in the future. First, facilitator’s participation was frequently impacted by instabilities such as illness and competing priorities, which are common in the lives of IDU, and must be planned for and navigated within the development and implementation of user-led projects. Other VANDU programs have addressed this issue by training multiple individuals for each role within a program, then arranging for these individuals to fill in for one another if someone misses their appointed duty. Second, although the facilitators spend a large amount of time on curriculum development, there was a greater need to develop their presenting skills before starting the campaign, and workshops involving detailed medical information would have benefited from the addition of a professional educator such as a nurse. Overall, the findings of this study indicate that greater efforts are needed to support existing user-led initiatives and to promote their growth and development as a means of providing education and services to IDU. Furthermore, health authorities and service providers developing services for IDU should incorporate the perspectives of IDU in service development and implementation to improve the relevance and cultural appropriateness of these services.

The present study has a number of limitations. First, the study focused exclusively on the perspective of individuals who were directly involved in the development and facilitation of IS Team education workshops. As such, the views presented by IS Team members may not be representative of the experiences of IDU involved in other interventions. Second, the perspective of one of the initial IS Team members was not captured because he withdrew from the team early in the development phase of this education campaign. This member withdrew to pursue another employment opportunity, although we have no reason to believe that his perspective would have been inconsistent with that of the remaining team members. Third, although IS Team members were told that their identity would be kept confidential and were encouraged to provide open and honest feedback on their participation, some participants may have been inclined to provide overly positive evaluations given their association with the campaign. However, an overly positive evaluation does not appear to be reflected in the findings, as facilitators provided numerous critiques of their own efforts and expressed a desire to improve and expand their existing activities to address additional issues facing local IDU. Finally, this study sought the feedback and impressions of workshop facilitators, yet it is also important to evaluate the perspective of the recipients of this education. While these data have been collected, we have elected to focus here on the experiences of facilitators and will present the findings related to the perspectives of recipients in a separate manuscript.

In conclusion, this study demonstrates the feasibility of involving IDU in educational initiatives targeting unsafe injecting. Our findings demonstrate that involving IDU in prevention activities improves relevance and cultural appropriateness of interventions while providing individual, social, and professional benefits to IDU directly involved in development and implementation of such interventions.

## Endnotes

^1^ Given that varied forms of pharmaceuticals require different preparation procedures, specific information was provided on how to prepare morphine eslon capsules, morphine kadian capsules, hydromorphone, talwin and ritalin, and methadone for injection.

## Abbreviations

IDU: People who inject drugs; VANDU: Vancouver area network of drug users; IS Team: Injection support team; DTES: Downtown eastside.

## Competing interests

The authors declare that they have no competing interests.

## Authors’ contributions

TK, WS, and CC conceived and designed the study. CC collaborated with the IS Team, attended all planning and development meetings related to the education campaign, observed all education campaign workshops, conducted all interviews, and actively sought team member’s opinions and feedback on research process. CC coded and analysed transcripts with input from IS Team members. RA assisted with analysis and interpretation of the data. TK and CC prepared the first draft of the manuscript. GC and WS assisted with the main content and provided critical comments on the final draft. All authors have read and approved the final version submitted for publication.
